# Stochastic Resonance Reveals “Pilot Light” Expression in Mammalian Genes

**DOI:** 10.1371/journal.pone.0001842

**Published:** 2008-03-26

**Authors:** Andrey Ptitsyn

**Affiliations:** Center for Bioinformatics, Department of Microbiology, Immunology and Pathology, College of Veterinary and Biomedical Sciences, Colorado State University, Fort Collins, Colorado, United States of America; University of Nottingham, United Kingdom

## Abstract

**Background:**

Microarrays are widely used for estimation of expression of thousands of genes in a biological sample. The resolution ability of this method is limited by the background noise. Low expressed genes are detected with insufficient reliability and expression of many genes is never detected at all.

**Methodology/Principal Findings:**

We have applied the principles of stochastic resonance to detect expression of genes from microarray signals below the background noise level. We report the periodic pattern detected in genes called “Absent” by traditional analysis. The pattern is consistent with expression of the conventionally detected genes and specific to the tissue of origin. This effect is corroborated by the analysis of oscillating gene expression in mouse (*M.musculus*) and yeast (*S. cerevisae*).

**Conclusion/Significance:**

Most genes usually considered silent are in fact expressed at a very low level. Stochastic resonance can be applied to detect changes in expression pattern of low-expressed genes as well as for the validation of the probe performance in microarrays.

## Introduction

Microarrays have become a standard technique in biological research. From the beginning, the focus in microarray experiments has been on taking the simultaneous snapshot of a large number of genes, rather than exact measurement of expression level for a small number of selected genes. Over the years the technology has undergone a significant evolution, allowing a reliable identification of functional relation of co-expressed genes and a good estimation of expression level for particular genes [1], [2]. However microarray performance is still limited by the background noise. Advanced normalization and summation algorithms[3], [4] can improve signal to noise ratio for the low-expressed genes. However, none of the contemporary algorithms can help with identifying expression of the genes expressed at such low level that the luminescent signal reading from the spot does not exceed the reading from the space between spots. It is commonly assumed that genes whose expression could not be identified by either microarray or RT-PCR experiment are silent. This assumption is intuitive, but not founded in biology. In contrast, most recent studies indicate the presence of eukaryotic transcription initiation complexes at the promoters of majority of “silent” genes for which no transcripts could be detected [5]. On the other hand, the situation where the signal to be detected is weaker than the ambient noise and could not be registered directly is not new in the other areas of science. The effect of Stochastic Resonance (SR) is well studied and widely applied in physics [6], [7] and even some areas of biology [8], [9]. Stochastic resonance is a counter-intuitive effect of amplification of a weak periodic signal by an increase of ambient noise (see Supplemental Figure S1). But what periodic signal in gene expression could be detectable with SR approach?

In 2006–2007 we have published a series of papers characterizing the oscillating patterns of gene expression in metabolically active peripheral tissues in mice. The circadian oscillation we reported extends far beyond the commonly accepted 10–15% of genes directly regulated by the circadian molecular clock [10], [11], [12], [13]. The accumulated evidence allows us to postulate that oscillation is a basic property of expression of all genes, not necessarily connected with any specific gene function. However, the oscillatory properties, such as phase and amplitude are dependent on the gene function and differ between tissues and experimental conditions [13]. Oscillatory patterns of expression in major housekeeping genes responsible for the energy balance (PPAR) and basic transcription (TBP) are bound to impose the same patterns on all transcribed genes regardless of the volume of transcription.

## Results and Discussion

**Figure 1:**
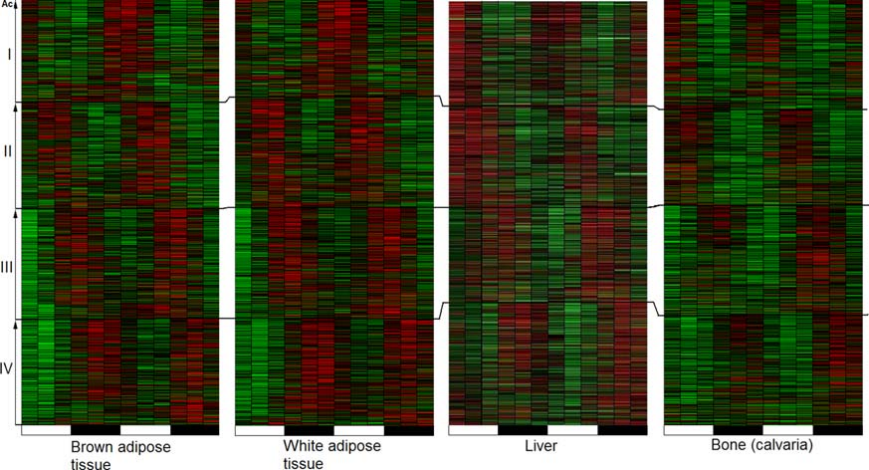
Circadian expression pattern in transcripts never called present. In spite of being considered silent most genes called absent are expressed in a daily changing pattern of elevated (red) and lowered expression level (green) over the 48h period of alternating light and darkness (bottom), consistent with circadian pattern in highly expressed genes. The plot shows four phase groups (roman numbers) in four murine tissues (data from [10] and [12]). On each pane expression profiles are stacked in order of autocorrelation with 24h lag (Ac, vertical axis).

The heat map plot in Figure 1 shows the pattern of circadian expression in approximately 30% of all genes interrogated by the Affymetrix mouse expression microarray. At first glance the pattern of two red (zenith) and two green (nadirs) areas over two-day period is remarkably similar to the previously published circadian expression patterns in mice [10],[14]. However in this case none of the genes selected for analysis has been called “Present” even once at any of the 12 time points. This effect is not specific to a particular tissue and observed in all mouse and yeast data sets considered in this paper.

**Figure 2:**
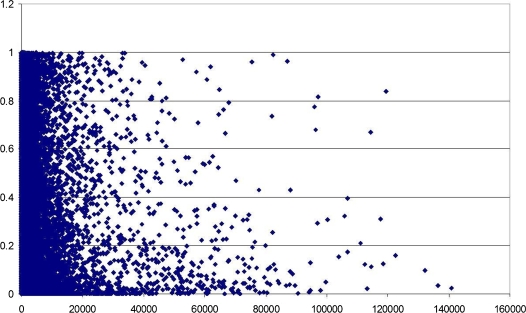
A scatter plot of mean intensity (axis X) and likeliness of periodicity estimated by Pt-test p-value (axis Y).

**Figure 3:**
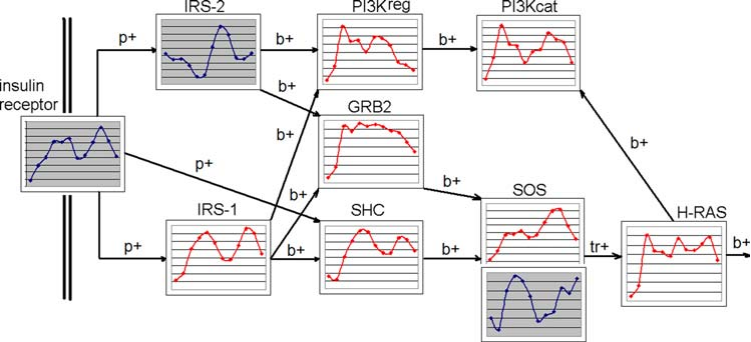
Insulin regulation of lipid metabolism in white adipose tissue (fragment). Transcripts called present are plotted with a red line on a white pane, absent transcripts are plotted blue on gray. Interactions are marked as p+ (activation by phosphorylation), b+ (activation by bonding) and tr+ (transcription activation). Timing of insulin receptor substrate transcription (IRS-1) is perfectly synchronized with insulin receptor transcription rate in spite of the absence call for the latter.

In most studies such “silent” genes are excluded from further analysis on the early stages. The filtration criteria are usually more stringent, selecting only genes called “Present” in at least half of all time points [15]. In the previous publications we have reported circadian oscillation in nearly 100% of all genes [13]. But the oscillating pattern does not show a strong dependence on the absolute level of expression or any regard to the signal/background noise ratio of the Affymetrix GeneChip. Figure 2 shows relations between likeliness of circadian oscillation (estimated by a periodicity test p-value) and the overall median of expression signal in time series. There is no indication of a threshold associated with presence or absence call. Genes expressed below the noise level (typically with signal reading under 150) generate the same pattern as highly expressed genes. This finding is corroborated by the results of periodicity tests performed on the subset of non-present genes (see Table 1). As expected, the number of “absent” genes for which a periodic pattern is observed with the confidence level of *p<0.1* is lower and the expression profiles are generally noisier compared to analysis of entire set of transcripts [13]. However, in spite of the lower signal to noise ratio the underlying baseline circadian, oscillation is detectable in majority of the profiles. This pattern and the proportion between phase groups are consistent with that of the “present” genes or the mixture of “present” and “absent” genes (transcripts). These observations lead to the conclusion that the criteria separating “present” from “absent” genes is arbitrary. The low signal emitted from the microarray probes can be below the noise level for the chip at each particular time point. However, it reflects the pattern of gene expression rather than an ambient noise. This point can be further illustrated by Figure 3 which shows expression pattern of transcripts called “Absent” and “Present” in the contexts of a biological pathway (a fragment of insulin signaling pathway, data from Metacore database, GeneGo Inc.). In spite of the absence call for the insulin receptor the white adipose tissue is known to respond to the insulin signal [16]. The profile for the “Absent” insulin receptor is pronouncedly circadian and perfectly synchronized with production rate of the insulin receptor substrate, which is called present.

**Table 1:** Numbers of circadially oscillating “absent” genes as reported by different algorithms and testing strategies.

Tissue	Number of probesets always absent	Fisher's g-test	Auto-correlation	Pt-test	Phase continuum method			
					g-test	Auto-correlation	Pt-test	KS fit for permuted periodogram
Brown adipose tissue	7170 (31.60%)	607	767	856	313	7130	7130	6817
White adipose tissue	5427 (23.92%)	514	620	640	303	5387	5387	5084
Liver	7570 (33.36%)	643	757	955	477	7530	7530	7049
Bone	5748 (25.33%)	478	536	1931	n/a	n/a	n/a	n/a

Periodic patterns observed in genes usually considered unexpressed are not necessarily associated with circadian rhythm. Analysis of respiratory oscillation pattern in *S.cerevisae* reveals a large group of genes (or rather transcripts interrogated by Affymetrix probe sets) that demonstrate a clear oscillating pattern consistent with that of highly expressed genes (Supplemental Figure S2).

Why do the “absent” genes, with expression pattern otherwise indistinguishable from the background noise suddenly show signs of expression consistent with that of reliably detectable genes? The explanation has been outlined in the abstract of the very first paper reporting the effect of SR [6]. The paper had shown that a dynamical system subject to both periodic forcing and random perturbation may show a resonance (peak in the power spectrum) which is absent when either the forcing or the perturbation is absent. In microarray gene expression studies the threshold is defined by the signal and noise levels estimated from the luminescent signal read from the spot with immobilized probes for a specific gene (transcript) and the background luminescence from the space between spots and/or blank spots with no specific probe. The details of signal detection may vary, particularly with Affymetrix summation algorithms (see [17] for review). However, regardless of the image processing and inference procedure microarray can be viewed as a detector with a certain threshold. All contemporary methods concentrate on improving the signal to noise ratio by either lowering the noise or amplifying the signal or both. However, the underlying periodicity of gene expression can provide an essential component for the SR to take effect. With known periodicity of the signal we have all necessary factors for SR. Periodicity of expression in nearly 100% of eukaryotic genes has been demonstrated in our recent paper [13]. The dominating rhythm is circadian in case of murine peripheral tissues. Expression pattern in yeast (*S. cerevisae*) is dominated by metabolic oscillation in respiratory cycle and this rhythm is also observed in nearly 100% of all genes (transcripts). However, the effect of SR can be achieved even without a natural baseline oscillation. Oscillation can be generated by repetitive application of perturbation (signal, treatment) in a biological system. Periodicity does not have to be time-wise. Regular placement of replicate probes on a lattice can also be viewed as a periodic signal across the surface of microarray and in combination with background noise it can create the effect of SR. In this case application of SR would require a new specifically designed microarray as well as significant modification of the analysis pipeline, starting from the image analysis on and between the spots of attached probes. In all cases the algorithm for detection of signal is based on a test for periodicity in a series of measurements rather than static comparison of signal and background noise levels.

Using the SR methodology, the test for gene silence can be formulated as follows: in presence of both periodic signal with known frequency *ω* and stochastic noise the null hypothesis (H_0_: gene Y is expressed) is equivalent to H_0_: expression signal for gene Y is periodic with frequency *ω*; H_0_ could not be rejected if test for periodicity is positive, alternative hypothesis (H_A_: gene X is silent) is accepted if there is no evidence of oscillation with frequency *ω*. There are a few available tests for periodicity; we suggest Pt-test [14], specifically developed for short time series with low sampling rate, typical for gene expression profiles. This test can be applied in conjunction with digital signal processing in phase continuum approach [13], which increases the test's ability to identify baseline oscillation. The concept of assigning detection calls using stochastic resonance is further illustrated by the computer program in supplemental materials (supplemental file Code S1). This program implements the simplest variant of detection call assignment: if sliding frame in phase continuum tests positive for baseline oscillation the genes found in this frame are called “Present”. More sophistication can be added by testing the presence of the baseline oscillation by a panel of statistical tests and/or taking in account the number of adjacent frames testing positive or negative. Multiple testing of FDR adjustment in not applicable for the reasons explained in the Methods section.

Detection of the extra-low gene expression has a few important implications. Long term practice of using microarray and RT-PCR technology has created a perception that a gene for which signal has the same intensity as ambient noise is not expressed. However, this fact relates to the resolution ability of the method rather than a real property of the gene. Using the principle of SR we greatly improve our ability to detect weak signals, but this method also has its limit. We observe expression of a large number of genes previously considered silent, but again this signal sinks into noise with no clear landmark separating expressed and silent fractions of genes. Could it be that the latter fraction does not exist and all genes are expressed, even at a miniscule rate? The entire concept of “silent” genes is created by our inability to detect extremely low transcript concentrations. There is no obvious landmark separating low-expressed genes and below-detection-threshold genes. Summing up the number of conventionally detected transcripts and transcripts detectable by SR leaves a very small fraction of truly silent gene candidates. This fraction also contains transcripts for which microarray probes are not performing as intended, which further reduces the number of potentially silent genes almost to none. Recent publication has already demonstrated that most human protein-coding genes are primed for transcription initiation, including those for which no transcripts could be detected [5]. Now we can detect those elusive transcripts with the new computational tools and a novel approach to the analysis of low-abundance transcripts. The “pilot light” suggested in the title seems to be more appropriate than “silent” for the genes expressed below the standard detection threshold. Such genes are likely to have transcription initiation complex in place, but no significant accumulation of mature transcripts in the cytoplasm. Theoretically, the concept of all genes being expressed, only at very different scale does not contradict the accumulated knowledge about cellular processes. However, ability to detect the extremely low expression and account for it in the experiment design opens new prospective for better, more complete understanding of the cellular processes, better account for potential adverse effects in medication and more precise biology in general.

## Materials and Methods

### Murine circadian expression data sets

We have completed independent circadian studies in AKR/J mice acclimated to a 12 hr light: 12 hr dark cycle, harvesting sets of 3–5 mice at 4 hr intervals in duplicates over a 24 hr period [10]. Total RNA samples from inguinal (iWAT) white adipose tissue, brown adipose tissue (BAT), and liver have been assayed by Affymetrix microarrays. A few genes have been selected for validation with RT-PCR for the expression profile of representative circadian rhythm genes in all 3 tissues. The transcriptomic data set contained over 22,000 gene expression profiles for each of 3 different tissues. In the current study, we have used only the murine liver data. Since each time point was sampled twice, the following Fourier transform for each profile can be re-arranged into a short time series that represents two complete circadian cycles. Profiles have been smoothened by a 3^rd^ degree polynomial procedure and median-subtracted. For better compatibility, the same smoothing and median subtraction procedure has been applied to all other data sets.

### Yeast data set

We have re-analyzed the time series data set provided by Dr. Tu and Dr. McKnight [18]. It has been reported that the time series covers approximately three periods of respiratory cycle and the majority of expressed genes follow this oscillating pattern.

### Algorithms

#### Data pre-processing

Profiles have been smoothened by a 3^rd^ degree polynomial procedure and median-subtracted. For smoothing we use seven-point Savitzky-Golay algorithm [19]. To take advantage of all points in the time series a single-pass smoothing has been applied in a circular manner, with the last points contributing to smoothing the starting points. For better compatibility, the same smoothing and median subtraction procedure has been applied to all data sets.

#### Spectral Analysis

For purposes of spectral analysis, consider a series of microarray expression values for gene *x* with *N* samples of the form
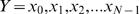
This series can be converted from time-domain, where each variable represents a measurement in time to a frequency domain using Discrete Fourier Transform (DFT) algorithm. Frequency domain representation of the series of experiments is also known as periodogram, which can be denoted by *I*(ω):
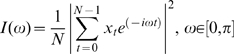
If a time series has a significant sinusoidal component with frequency ω∈[0, *π*], then the periodogram exhibits a peak at that frequency with a high probability. Conversely, if the time series is a purely random process (a.k.a “white noise”), then the plot of the periodogram against the Fourier frequencies approaches a straight line [20].

#### Fisher's g-test

The significance of the observed periodicity can be estimated by Fisher *g*-statistics, as recently recommended in [21]. Fisher derived an exact test of the maximum periodogram coordinate by introducing the *g*-statistic
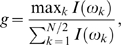
where *I*(*ω_k_*) is a *k-*th peak of the periodogram. Large values of g indicate a non-random periodicity. We calculate the *p*-value of the test under the null hypothesis with the exact distribution of *g* using the following formula:
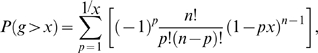
where *n* = [*N*/2] and *p* is the largest integer less than 1/*x*.

This algorithm closely follows the guidelines recommended for analysis of periodicities in time-series microarray data [21] with the exception that we applied a locally developed C++ code instead of R scripts.

#### Autocorrelation

For a given a discrete time series *Y* = *x*_0_, *x*_1_, *x*_2_,… *x_N_*_−1_ the autocorrelation is simply the correlation of the expression profile against itself with a frame shift of *k* data points (where 0≤*k*≤*N*−1, often referred as the lag). For the time shift *f*, defined as *f* = *i*+*k* if *i+k<N* and *f* = *i*+*k*−*N* otherwise
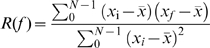
For each time series we calculate the maximum positive *R(f)* among all possible phase shifts *f* and use tabulated 0.05 significance cutoff values for correlation coefficient. Time series that shows significant autocorrelation *R(f)* with the lag *f* corresponding to one day (6 time points) are considered circadially expressed.

#### Pt-test

Consider a time series *Y* = *x*_0_, *x*_1_, *x*_2_,… *x_N_*_−1_ in which technical variation approaches or even exceeds the amplitude of periodic expression. In a very short time series stochastic noise often obscures periodicity. However, the periodic change of the base expression level can still be identified in spite of the high noise level. If the periodogram of the original time series *IY(ω)* contains a significant peak corresponding to a particular frequency (for example, circadian) this peak results from observation is the *Y*. A random permutation would preserve the same noise level, but not the periodicity. Let *YR* be a random permutation of the time series *Y.* Its corresponding periodogram is *IR(ω)*. After DFT a periodogram *IR(ω)* would represent only the peaks occurring by chance. However it will miss the true periodic frequencies unless permutations happen to preserve the period, for example if the rank of each point *x* in permutated series *YR* is equal *x_Y_*±n*p where *n* is a natural number and *p* is a period corresponding to a significant peak in *IY(ω)*. To avoid random re-institution of periodicity we generate *YR* by multiple shuffling of randomly selected time points *x_n_*⇔*x_m_*, where |*n*−*m*|≠*p*, i.e. each shuffle is swaps time points from different phase. Comparing permutations with deliberately wiped out periodicity to the original time series we can estimate whether a particular order of observations (i.e. time series) is important. For each gene expression profile we generate two series of *min(n!,100)* random permutations. Each permutated series *YR* is transformed to the frequency domain and a single peak of the periodogram *IR(ω)* is stored. The p-value for the null-hypothesis of random nature of a particular peak of periodogram can be estimated by comparing the stored *IR(ω)* values to the observed *I(ω)*: 
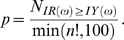
High *p*-value exceeding the threshold, for example 0.05, means that at least 5 out of 100 random permutations of time series produce a periodogram with the same or higher peak, corresponding to a given periodicity. Low *p*-values indicate a significant difference between periodogram *IR(ω)* preserving circadian periodicity and randomly permutated periodogram *IY(ω)* with the same level of technical variation. This difference leads to rejection of the null-hypothesis of purely random nature of variation in the original time series *Y*.

#### Phase continuum

We start with phase classification, assigning each gene a phase based on maximal correlation to an ideal cosine curve. This method is superior to assigning a phase by position of peaks only because it takes into account more data. Each profile is subjected to z-score transformation equalizing the variation between time points. For each profile autocorrelation with circadian lag (*R_c_*) is calculated and all profiles are sorted first by phase then by descending order of *R_c_*. Concatenating all profiles of the same phase with equalized range of variation (amplitude) we generate a continuous stream *C_ph_* of measurements containing a clear signal on one end and stochastic noise on the other. This continuum is treated with low-pass frequency filter and polynomial smoothing. We analyze each phase fraction separately to detect the point at which circadian signal deteriorates beyond p = 0.05 significance cutoff. A window W moving along the stream is transformed to frequency domain using Discrete Fourier Transform (DFT). The resulting periodogram *I_w_* is compared a periodogram of a randomly permutated *W_r_* using Kolmogorov-Smirnov goodness of fit test. Once the point at which *I_w_* does not differ significantly from a random periodogram *I_wr_* is detected, we count all original gene expression profiles that have circadian signal above the established cutoff [13].

#### False Discovery Rate analysis

This methodology often applied to reduce the number of false-positive tests is based in the assumption of independent or mildly dependent [22] hypothesis testing. However, in case of testing timeline expression profiles for periodicity independence could not be assumes for a number of reasons. First, the pattern of circadian oscillation is obvious in the great majority of expression profiles as seen on heatmaps (Figure 1, for example). Second, analysis of correlation with phase shift (also used to identify phase groups) confirms high correlation of nearly all profiles to common cosine curves. Third, living cells are known to have more than one oscillator, but these oscillators are normally synchronized to the rhythm of the circadian molecular clock, active in peripheral tissues. Testing individual expression profiles for periodicity we are looking for manifestation of the same factor, hence not independent hypothesis. For these reasons FDR correction has not been applied to reduce the number of detected oscillating genes.

## Supporting Information

Figure S1 Illustration of the principal of stochastic resonance. A periodic signal could be too weak to be detected by existing methods (A). The threshold for a detector can be selected so that a stochastic ambient noise is occasionally detected. The occurrence of stochastic noise exceeding the detection threshold is non-periodic (B). However, in presence of both stochastic noise and a weak periodic signal (C) there is a higher probability for the noise to be registered above threshold. Consequently, the occurrence of noise exceeding threshold becomes periodic and detection of this periodicity indicates the presence of baseline periodic signal.
(0.04 MB DOC)

Figure S2 Heatmap of 323 transcripts (Affymetrix probesets) never called present at any time point. YG_S98 Affymetrix microarray has much fewer probesets compared to mouse expression arrays and almost all of these genes are found “present” or at least “marginal” at least once over the three periods of respiratory cycle. Only 323 genes are never called present. Pt-test indicates significant baseline oscillation with 3 periods within all profiles (36 time points each), although the pattern of 3 red zones is not obvious in all profiles. Only 3 transcripts show significant oscillation by fisher's g-test (p<0.1). These profiles are depicted on Supplemental Figure 3.
(0.12 MB DOC)

Figure S3 Expression profiles of the tree S.cerevisae probesets with p<0.1 by both Pt-test and Fisher's g-test. All three are never called present at any single time point. AFFX-BioDn-5_st represents a control sequence of bacterial origin which should not be present. Oscillating pattern of this probeset may be caused by a small contamination of the yeast culture with E.coli or cross-hybridization from the nearest yeast homologues of dethiobiotin synthetase. Two other probesets (6617_at and 7889_at) are annotated as “dubious ORF” and “nonessential protein”.
(0.09 MB DOC)

Code S1 This is an abbreviated C++ program that illustrates the concept of detection call based on the principle of Stochastic Resonance.
(0.41 MB ZIP)
