# Supplementary material for: Stochastic Resonance Reveals “Pilot Light” Expression in Mammalian Genes
Source: PLoS One. 2008 Mar 26;3(3):e1842. doi: 10.1371/journal.pone.0001842 (PMC2266998; doi:10.1371/journal.pone.0001842)
Supplement: Figure S1 — Illustration of the principal of stochastic resonance. A periodic signal could be too weak to be detected by existing methods (A). The threshold for a detector can be selected so that a stochastic ambient noise is occasionally detected. The occurrence of stochastic noise exceeding the detection threshold is non-periodic (B). However, in presence of both stochastic noise and a weak periodic signal (C) there is a higher probability for the noise to be registered above threshold. Consequently, the occurrence of noise exceeding threshold becomes periodic and detection of this periodicity indicates the presence of baseline periodic signal. (0.04 MB DOC) [file pone.0001842.s001.doc]

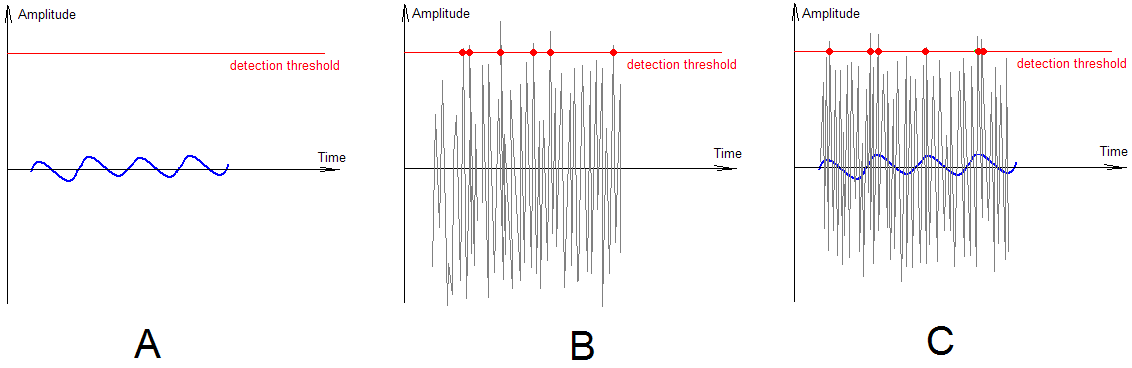


Supporting Figure S1. Illustration of the principal of stochastic resonance. A periodic signal could be too weak to be detected by existing methods (A). The threshold for a detector can be selected so that a stochastic ambient noise is occasionally detected. The occurrence of stochastic noise exceeding the detection threshold is non-periodic (B). However, in presence of both stochastic noise and a weak periodic signal (C) there is a higher probability for the noise to be registered above threshold. Consequently, the occurrence of noise exceeding threshold becomes periodic and detection of this periodicity indicates the presence of baseline periodic signal.
