# Supplementary material for: Stochastic Resonance Reveals “Pilot Light” Expression in Mammalian Genes
Source: PLoS One. 2008 Mar 26;3(3):e1842. doi: 10.1371/journal.pone.0001842 (PMC2266998; doi:10.1371/journal.pone.0001842)
Supplement: Figure S2 — Heatmap of 323 transcripts (Affymetrix probesets) never called present at any time point. YG_S98 Affymetrix microarray has much fewer probesets compared to mouse expression arrays and almost all of these genes are found “present” or at least “marginal” at least once over the three periods of respiratory cycle. Only 323 genes are never called present. Pt-test indicates significant baseline oscillation with 3 periods within all profiles (36 time points each), although the pattern of 3 red zones is not obvious in all profiles. Only 3 transcripts show significant oscillation by fisher's g-test (p<0.1). These profiles are depicted on Supplemental Figure 3. (0.12 MB DOC) [file pone.0001842.s002.doc]

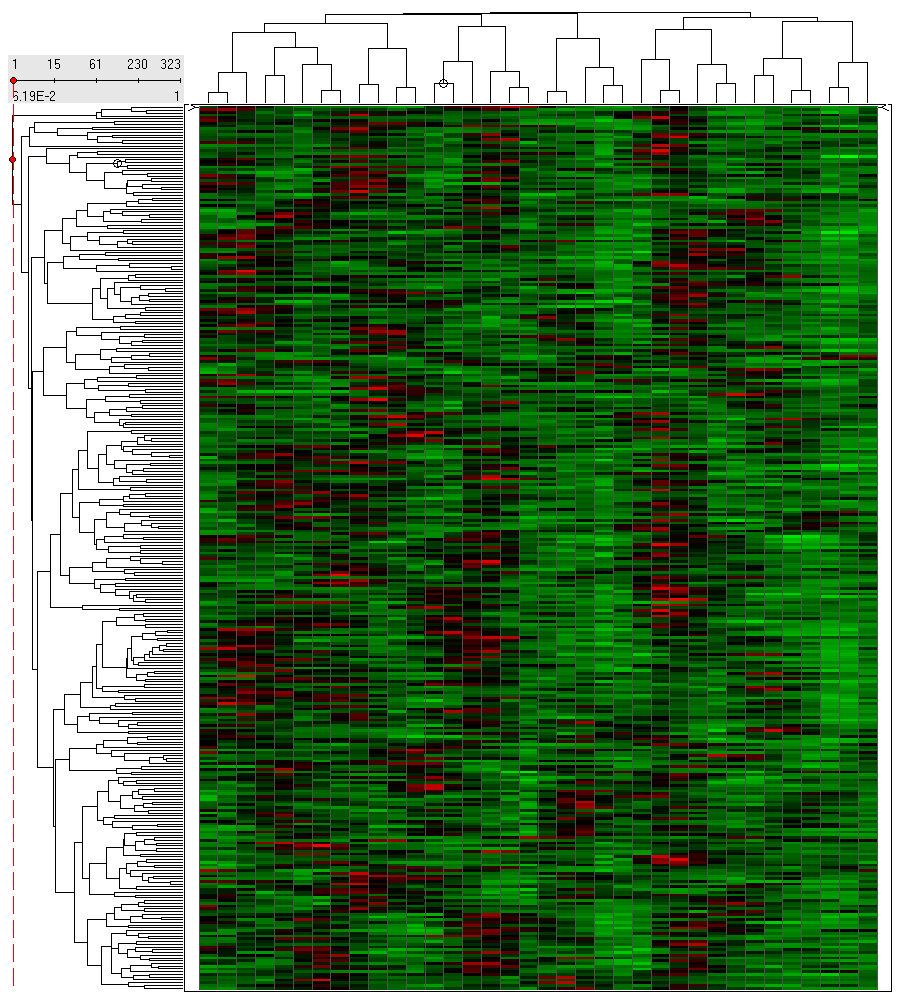


Supporting Figure S2. Heatmap of 323 transcripts (Affymetrix probesets) never called present at any time point. YG_S98 Affymetrix microarray has much fewer probesets compared to mouse expression arrays and almost all of these genes are found “present” or at least “marginal” at least once over the three periods of respiratory cycle. Only 323 genes are never called present. Pt-test indicates significant baseline oscillation with 3 periods within all profiles (36 time points each), although the pattern of 3 red zones is not obvious in all profiles. Only 3 transcripts show significant oscillation by fisher’s g-test (p<0.1). These profiles are depicted on Supporting Figure S3.
