# Supplementary material for: Stochastic Resonance Reveals “Pilot Light” Expression in Mammalian Genes
Source: PLoS One. 2008 Mar 26;3(3):e1842. doi: 10.1371/journal.pone.0001842 (PMC2266998; doi:10.1371/journal.pone.0001842)
Supplement: Figure S3 — Expression profiles of the tree S.cerevisae probesets with p<0.1 by both Pt-test and Fisher's g-test. All three are never called present at any single time point. AFFX-BioDn-5_st represents a control sequence of bacterial origin which should not be present. Oscillating pattern of this probeset may be caused by a small contamination of the yeast culture with E.coli or cross-hybridization from the nearest yeast homologues of dethiobiotin synthetase. Two other probesets (6617_at and 7889_at) are annotated as “dubious ORF” and “nonessential protein”. (0.09 MB DOC) [file pone.0001842.s003.doc]

-2

-1.5

-1

-0.5

0

0.5

1

1.5

2

2.5

1

3

5

7

9

11

13

15

17

19

21

23

25

27

29

31

33

35

AFFX-BioDn-5_st

6617_at

7889_at

Amplitude

Time

Supporting Figure S3. Expression profiles of the tree S.cerevisae probesets with p<0.1 by both Pt-test and Fisher’s g-test. All three are never called present at any single time point. AFFX-BioDn-5_st represents a control sequence of bacterial origin which should not be present. Oscillating pattern of this probeset may be caused by a small contamination of the yeast culture with E.coli or cross-hybridization from the nearest yeast homologues of dethiobiotin synthetase. Two other probesets (6617_at and 7889_at) are annotated as “dubious ORF” and “nonessential protein”.
